# Assessing Patient Engagement in Health Care: Proposal for a Modeling and Simulation Framework for Behavioral Analysis

**DOI:** 10.2196/30092

**Published:** 2021-12-08

**Authors:** Athary Alwasel, Lampros Stergioulas, Masoud Fakhimi, Wolfgang Garn

**Affiliations:** 1 Surrey Business School University of Surrey Guildford United Kingdom; 2 Management Information Systems Department College of Business Administration King Saud University Riyadh Saudi Arabia; 3 Data Science Research Group Faculty of Information Technology and Design The Hague University of Applied Sciences The Hague Netherlands

**Keywords:** modeling and simulation, behavioral analysis, patient engagement, behavioral factors, health care, human factors, outcomes, patient health, health policy, chronic diseases, behavioral model

## Abstract

Human behavior plays a vital role in health care effectiveness and system performance. Therefore, it is necessary to look carefully at the interactions within a system and how a system is affected by the behavioral responses and activities of its various components, particularly human components and actions. Modeling patients’ engagement behaviors can be valuable in many ways; for example, models can evaluate the effects of therapeutic interventions on health improvement, health care effectiveness, and desired outcomes of changing health lifestyles. Modeling and simulation (M&S) help us to understand the interactions within a whole system under defined conditions. M&S in patient behavior analysis involve models that attempt to identify certain human behaviors that most likely have an impact on health care operations and services. Our study’s overall aims are (1) to investigate the impacts of patients’ engagement and various human behavior patterns on health care effectiveness and the achievement of desired outcomes and (2) to construct and validate a framework for modeling patient engagement and implementing and supporting patient management best practices, health policy-making processes, and innovative interventions in health care. We intend to extract routinely collected data of different parameters from general patients diagnosed with chronic diseases, such as diabetes. Our plan is to design data sets and extract health data from a pool of >4 million patient records from different general practices in England. We will focus on the primary electronic medical records of patients with at least 1 chronic disease (>200,000 records). Simulation techniques will be used to study patient engagement and its impact on health care effectiveness and outcome measures. The study will integrate available approaches to develop a framework for modeling how patients’ behaviors affect health care activities and outcomes and to underline the characteristics and salient factors that operational management needs to be aware of when developing a behavioral model for assessing patient engagement. The M&S framework, which is under development, will consider patient behavior in context and the underlying factors of human behavior with the help of simulation techniques. The proposed framework will be validated and evaluated through a health care case study. We expect to identify leading factors that influence and affect patient engagement and associated behavioral activities and to illustrate the challenges and complexities of developing simulation models for conducting behavioral analyses within health care settings. Additionally, we will assess patients’ engagement behaviors in terms of achieving health care effectiveness and desired outcomes, and we will specifically evaluate the impacts of patient engagement activities on health care services, patient management styles, and the effectiveness of health interventions in terms of achieving the intended outcomes—improved health and patient satisfaction.

**International Registered Report Identifier (IRRID):**

PRR1-10.2196/30092

## Introduction

### Background

Behavioral analytics is the study of behavioral aspects through operational research methods for modeling, problem solving, and decision-making support [1]. Behavioral patterns can influence problem solving and policy making and enable modelers to mimic and study real-world situations.

Human behavior can alter the performance of any system with multi-stakeholder processes. An overall analysis of human behavior entails models of an entity, social interactions, and connections within a system [2]. It is therefore essential to incorporate human behavioral factors into the modeling of complex systems, such as health care.

Modeling and simulation (M&S) have been shown to be suitable methods for mimicking real-world scenarios and assisting researchers and practitioners in the manufacturing field and various other sectors [3,4]. M&S techniques lend themselves to the conceptual representation of the system at hand and its implementation via a computer model. By applying suitable methods in studies, M&S methods are used to represent different settings and real-world situations and determine the results of a specific state.

M&S have been shown to be useful methods for addressing health care—a complex and uncertain system involving a range of emergent behaviors that occur daily. Health care systems involve various actors and stakeholders and include diverse policy makers and practitioners, such as health sector regulators, patients, nurses, and physicians [5]. The features of M&S can be applied to health care systems to assess interventions and outcomes by incorporating human behaviors and human factors.

A wide range of M&S applications for enhancing ongoing developments in policy making and services continue to emerge. For instance, the use of the hybrid modeling approach has been shown to support short-term decision-making in emergency health care by helping emergency service facilities avoid overcrowding [6]. In the case of stroke care and within the context of precision medicine for stroke, a hybrid modeling approach has been shown to be the most beneficial [7]. The application of hybrid modeling has also been observed in the field of cardiovascular diseases; hybrid modeling approaches have been used to assess integrated care for challenging and complex diseases to offer comprehensive tools for decision-making [8].

A combination approach of using simulation and machine learning techniques, for example, has also been found to aid policy makers in designing care pathways and developing effective health care policies. The purpose of this approach is to use machine learning to provide valuable support in the development of simulation models [9]. Moreover, machine learning–based extractions, along with digital health tools and electronic health records, can improve health care providers’ communications in the provision of precision care and services.

M&S aim at providing modelers with tools for assessing system performance under different conditions via a top-down approach and can be helpful in predicting behaviors and possible outcomes. Furthermore, M&S can offer modelers the ability to mimic real-world problems, for which the derivation of causal relationships is vital [10]. M&S have been shown to have the potential to improve health care services in several health care areas, such as health risk assessment and cost-benefit analysis and policy evaluation [11]. Moreover, combining analytical methods and simulation techniques can be useful in studying the effects of lifestyle risk factors, assessing the quality of services, and predicting lifestyle risk behaviors linked to prevalent chronic diseases [12,13].

The M&S of such health data are conducted to identify associations among risk factors and promote selective targeting and the use of behavioral interventions among health care providers. However, the interpretation of such models might not be easy if too many variables are involved, and modelers may choose to simplify their approach in the early stages of model development to gain a full understanding of the problem under study.

Different simulation approaches have been incorporated to study the effect of population changes on the demand for health care services [14]. Thus, simulation models for behavioral analytics can be used in the analysis of patient behavior to enhance decision-making and improve health care effectiveness (ie, with the help of hybrid approaches).

Modeling human behavior involves considering different activities within the system under study, primarily activities related to cognitive processes and systems’ dynamic evolution (ie, mental activities resulting from the time-dependent interactions of humans and related activities) [4]. When modeling a system, such dynamic interaction needs careful assessment to ensure that human factors are weighted with external factors.

Brailsford and Schmidt [15] carried out research on incorporating human behavior in models of health care systems by using an M&S approach. They illustrated how simulation had been used to paint a picture of all of the parameters that contributed to the prevalence of disease by monitoring patients’ behaviors, including individuals’ characteristics. Furthermore, in their field study, which analyzed infectious diseases and infection control, Talib et al [16] noted that the spread of infections and diseases, such as HIV infection and AIDS, does not solely rely on physiological factors but also relies on behavioral aspects. This explains why it is essential to not ignore human behavior when developing models and applying M&S methods. Subsequently, if behavioral factors are overlooked, simulation results may not do much to help researchers and practitioners understand real-world situations.

When applying behavioral analysis approaches to model development, representations of various components of the system under study can provide insights for understanding how the system's various factors are interconnected and how it responds to changes over time. M&S methods have been typically used to account for social, economic, and environmental changes, as modeling provides insights into system behaviors and possible outcomes.

In general, M&S methods comprise evidence-based techniques and tools that can provide important insights toward gathering the evidence required to improve overall performance and aid policy settings, including sectors like health care [17]. M&S have been used to assess risk factors, including human factors, and to conduct behavioral analyses for establishing the interdependencies that exist among various components of complex systems via a process that focuses on interconnectivity and changes that occur over time [1,18-20].

In the health care sector, M&S have been effectively used to study crucial public health issues [21-23]. It has become evident that using M&S for behavioral analytics can be key in enabling modelers to recommend service improvements, of which the results can be directly reflected by patients’ satisfaction and well-being. Our research focuses on the application of M&S to the analysis of patient behaviors within health care systems and aims to demonstrate how this method can help with the evaluation of health care effectiveness for suggesting service improvements.

### Objectives

Patient engagement pertains to patients being active in decisions and actions related to their own health and preferences toward enhancing their satisfaction with the health care system and improving health care outcomes. In health care decision-making, patient engagement is described as the approach whereby health care staff members engage with patients as equal partners to make healthy choices based on the best health care evidence available and patients’ informed preferences and care expectations [24]. The experience of engagement is a key qualifier of the exchanges between patients and their health care providers [25].

The overall goal of our study is to identify the leading factors underpinning patient engagement activities to achieve a proper understanding of the relationship between patient engagement experiences and health care effectiveness in terms of achieving the desired outcomes of health interventions and healthy lifestyles. Gauging the behavioral aspects of patients’ engagement or disengagement activities can contribute to showing how patients respond to care plans and the delivery of health care interventions and can therefore help with optimizing health care outcomes. Hence, the main objectives of our study are as follows:

- To study the impact of patients’ engagement behaviors on health care effectiveness and desired health outcomes
- To identify factors that drive and influence engagement and disengagement in patients with chronic diseases (ie, with a focus on diabetes)
- To develop a generic M&S framework for patient behavior analysis

As one of the most prevalent chronic diseases, diabetes can have long-term effects and induce profound changes to patients’ lifestyles and behaviors. Patients with chronic diseases tend to exhibit various engagement behaviors and practice different lifestyles. Thus, they may or may not be willing to follow and be involved in all necessary actions within their care management plans. Therefore, our study sets out to use the M&S framework for behavioral analysis and incorporate human behavior in modeling to further demonstrate how patients’ engagement behaviors may influence health care effectiveness and desired health care outcomes.

## Methods

### Study Type

The provided data will be analyzed to identify the leading behavioral factors of patient engagement and determine how engagement behaviors can affect health care effectiveness and the desired outcomes of following health care plans for chronic disease management and treatment, such as diabetes care plan outcomes. The following aims will be put forward: (1) to identify the causal influences and interdependencies in relationships between patients’ behaviors and health care effectiveness and between patients’ behaviors and the desired outcomes of following required healthy activities and (2) to evaluate the association between patients’ engagement patterns and the achievement of effective patient care management and patient satisfaction. A hybrid M&S approach will be used to study patient behavior in the highly complex health care system and inform health care practitioners and stakeholders.

Appropriate and widely used M&S methods will be used to explore interdependencies among system parameters. M&S models have so far been used in health care systems to primarily analyze specific long-term diseases [26], virus spread and transmission [27,28], patient flow in accident and emergency (A&E) departments [29,30], and various policy evaluations [31,32], and little attention has been paid to behavioral factors and their influence. We intend to conduct M&S to potentially identify leading factors that influence and affect patient behavior and engagement.

The proposed hybrid M&S approach will involve the combination of qualitative system dynamics (QSD) with M&S techniques and be used to develop a generic hybrid M&S framework for behavioral analysis in health care. The hybrid M&S approach will be used in M&S framework development to help provide a deeper understanding of how human behavior affects health care outcomes in models. QSD can represent specific system dynamics and mediate the qualitative expression of system dynamics mechanisms and any possible quantitative representations.

System dynamics modeling will be used as a simulation technique to explore interdependencies among parameters. System dynamics modeling is a simulation method that applies to dynamic problems that arise in complex systems. It is one of the main techniques for analyzing complex systems and problems, as it allows modelers to understand the interfaces between system components and information feedback that show dynamic behaviors within a system [33,34].

A systems approach will be implemented to study the behavioral analysis of complex systems and inform health care policies and strategies. The simulation technique assumes that challenging behaviors within a system result from accumulations of people, information, and psychological states, and the technique is meant for reinforcing feedback mechanisms [35]. It incorporates the feedback processes that unfold over time to determine the dynamics of a system.

System dynamics models have been used in health care systems to analyze specific chronic diseases, the transmission of diseases, patient flow in A&E departments, and policy evaluation and decision support systems. System dynamics explores behaviors within the social systems of an organization, and systems dynamics modeling can be conducted to model the flow of each agent rather than individual agents’ behaviors. Compared to other simulation methods, system dynamics modeling works at a more collective level to prove how organizational, human, and social structures and interactions influence the behavior of systems.

System dynamics has been applied in various behavioral analytics studies related to health care. It can help health care decision makers gain a deeper understanding of public health issues, such as the spread of an infectious disease [36,37]. It has also been used to analyze patient behaviors and unhealthy lifestyles that tend to contribute to major public health problems at the societal level [38,39]. Principally, system dynamics has been useful for incorporating behavioral factors as part of the feedback that affects decision-making in health care settings [40].

We intend to use a hybrid M&S approach, and the M&S framework for behavior analysis will use system dynamics modeling as one of the simulation techniques to focus on viewing patients as aggregates with common characteristics and to potentially identify the leading factors that influence and affect patient behavior and engagement. The study intends to integrate the hybrid M&S approach to construct a model for studying behavioral changes. Stock-and-flow diagrams based on defined conceptual models will be created for illustrative purposes, and inputs from available data sources will be integrated into the model.

Agent-based simulation (ABS) can also be valuable in analyzing individual behaviors from large health care data sets. After evaluating the developed M&S framework as an instrument of guidance that modelers can use for incorporating human behavior, more work and future studies will be carried out to use other simulation techniques, such as ABS and discrete-event simulation (ie, when the initial evaluation of the framework is done and large data sets become available). This will encourage modelers and practitioners to incorporate behavioral characteristics per the framework guidance.

### Study Design

Our exploratory study will use M&S methods to investigate factors that influence patient engagement in chronic disease care, specifically engagement in diabetes care. We will examine various aspects of behavioral engagement activities, such as outpatient attendance, patients’ adherence to medication, compliance in terms of follow-up visit adherence, A&E department attendance, and bed occupancy. The study will focus on engagement behaviors during diagnosis and treatment and factors that can result in better patient engagement to ensure the effectiveness of health care.

Patient engagement is characterized by various closely connected aspects related to cognitive, behavioral, emotional, and mental factors. The study will assess the behavioral aspects of engagement that may not be totally separated from other aspects in an attempt to shed light on how to link such aspects with operational and clinical factors within health care settings.

Textbox 1 provides a list of tentative variables that may be associated with patients and health data set records. Simulations based on a hybrid approach and a combination of QSD and system dynamics modeling will be conducted to evaluate different engagement patterns and strategies based on desired outcomes and health care effectiveness.

**List of possible patients’ various variables to be extracted from the data sets.**

**Demographic variables**

- Age, ethnicity, gender, education, occupation, income range, employment, and postcode

**Mental health variables**

- Psychotropic medications, antidepressant prescriptions, antidepressant scripts, issued sick notes, and sick certificates

**Chronic disease variables**

- Height, weight, systolic and diastolic blood pressure, temperature, and diabetes

**Behavioral and operational variables**

- Number of appointments, wait time, arrival time, length of stay, immunization and vaccination status, screening, counseling, medication adherence, antidepressant adherence, antidepressant scripts, refill prescriptions, access to treatment, treatment adherence, session adherence, therapy attendance, health care visit adherence, self-management behavior, care seeking, number of disease episodes, the course of therapy completed, number of admissions, inpatient admission (number of occupied bed days), accident and emergency department attendance, outpatient attendance, bed occupancy, number of bed days, test results, hospitalization rates, and recovery rates

### Sample Size and Power Calculation

It is estimated that the sample of patient records (ie, those of patients with at least 1 specific chronic disease, such as diabetes) will consist of >200,000 of the >4 million available records of general patients. With this sample size, a high statistical confidence in the results is expected.

### Study Population

It is anticipated that the study population will pertain to >4 million records of general patients aged ≥16 years, and >200,000 records are expected to pertain to patients with at least 1 specific chronic disease, such as diabetes.

### Exposures

There is no treatment to be offered in the study. However, data related to general treatment and medication adherence will be analyzed as part of the study.

### Outcomes

The study will identify the leading factors that influence and affect patient engagement and the interdependencies between patient engagement and the effectiveness of health care management plans. We will report on the following outcomes:

- The identification of the leading factors of patients’ engagement behaviors
- A demonstration of how the incorporation of human behavior and actions in modeling would help modelers achieve a better representation of complex issues in health care and reflect on the desired outcomes of health care interventions
- Suggestions for improving health care management plans

### Use of Any Linked Data and Plans to Link Data

We plan to use the data sets described by de Lusignan et al [41]. Their source is the Research and Surveillance Centre of the Royal College of General Practitioners—a collection and network of general practitioners that cover the UK population. We plan to use the primary care electronic medical records of patients with chronic diseases, such as diabetes.

The primary care data collected from several general practices will be linked by using the Secure and Private Record Linkage (SAPREL) method. The SAPREL method allows for the linking of patients’ records without the need to know patients’ identities [41] and therefore allows for the collection of general patients’ variables, including various physical and mental variables. For example, in a study by de Lusignan et al [41], the authors calculated the number of issued prescriptions as a proxy measure of medication adherence, as collecting only a single script without conducting further follow-ups would be a big problem. Furthermore, the authors suggested that more careful studies would be needed to gauge patient engagement and its relationship with clinical, operational, and mental variables, such as bed occupancy and outpatient attendance [41].

At this stage, there are no plans for involving patients or user groups. However, at a later stage and on the basis of study outcomes, we may be required to consider this for further research.

### Data and Statistical Analysis Plan

We will make use of an M&S behavioral analysis approach and use real health care data sets of patients with a chronic disease diagnosis to conduct a real-world case study that uses the developed M&S framework for behavioral analysis. The patients will be classified based on their patterns of engaging or not engaging with the required actions in the health care management plans provided to them. Hypotheses of causal influences will be formulated to compare behavioral patterns and gain insights into the impacts of different behaviors on health care operations. The model will compare different simulated behavior patterns over time in relation to the health data variables of patients.

## Results

We anticipate that we will finish the extraction of data and the analysis of results by March 2022. After completing the M&S study, the dissemination of results will occur in due course. The model will be used for the evaluation and validation of the M&S framework under development. The findings will be published in conference proceedings and peer-reviewed journals related to M&S applications in health care management and primary health care informatics.

We also anticipate some limitations to our study. In relation to data sources, possible limitations are foreseen to pertain to the availability of well-defined behavior parameters and the nature of data related to patients’ adaptability, engagement, and communication skills, which vary among patients. Other limitations could be related to the very nature of models and simulations in the health care sector, as health issues may arise from the interactions of a relatively large number of parameters. Therefore, a health system is not constrained to patients' personal and unique characteristics and is highly complex [42].

To study how behavioral analysis can be linked to health care effectiveness and service enhancements in health care management, general patients’ variables, including demographic, physical, mental, and behavioral aspects, will be collected. Chronic diseases are embedded in the complex connections and interactions among the multiple and diverse variables that add to health care–related challenges [43]. Some data are expected to be missing or incomplete for some patients (eg, some variables related to follow-up visits may not exist).

## Discussion

Our study aims to analyze and model the leading factors of patients’ engagement behavior patterns in health care settings and the impacts of patients’ engagement behaviors and to understand how patients’ engagement behaviors can be linked to health care effectiveness in terms of achieving desired outcomes.

It has been argued that behavioral analysis, which is the study of human behavior and its impacts on systems and processes, can result in the increased effectiveness of health care interventions and policy making improvement [44]. Most traditional health care models rarely detail the precise impacts of behavioral reactions and interactions or the dynamics of these reactions and interactions. This indicates the need to construct models that more explicitly incorporate behavioral aspects to modulate the dynamics of health care systems. Such an aim can be achieved by applying a generic M&S framework with proper M&S techniques to the incorporation of human factors to understand the impacts of various patient engagement activities on health care effectiveness in terms of achieving desired health outcomes.

Our framework seeks to provide specific guidelines for incorporating human behavior in hybrid M&S studies that take into consideration underlying factors and their influence and can possibly benefit from a mix of M&S methods [45,46]. The choice of using a simulation technique combined with operations research methods might help with examining and understanding the effect of incorporating human behavior and how to benefit from modeling to mimic real-world situations. Although the focus of our study is on evaluating the hybrid system dynamics–QSD framework, which uses a system dynamics technique to view patients as aggregates, the first step of this evaluation should be assessing the framework’s understanding of the possible effects of human behaviors and actions. For future research, the applications of ABS QSD or discrete-event simulation QSD in behavioral analytics will also be investigated.

As previously stated, our main focus will be on the behavioral analysis of patients diagnosed with at least 1 chronic disease, such as diabetes. The data sets described by de Lusignan et al [41] will be used in our study to gain insights into patients’ behavioral patterns. The study intends to identify patients’ engagement behavior variables and intends to use and evaluate the M&S framework for behavioral analysis via simulation models.

Our goal is to guide modelers in developing approaches to tackling complex problems that will help shape processes, interventions, and policies for improving health care effectiveness and operations and, possibly, behavior change strategies. This is one of the necessary steps for incorporating behavioral analysis in the tackling of problems involving human actions, particularly those within health care settings. Once the framework has been evaluated and behavioral analysis has been incorporated into simplified health care environments, modelers will acquire the know-how for incorporating behavioral analysis into the assessment of human activities and the tackling of severe, worldwide health problems, such as the COVID-19 pandemic.

## Abbreviations

A&E: accident and emergency
ABS: agent-based simulation
M&S: modeling and simulation
QSD: qualitative system dynamics
RCGP: Royal College of General Practitioners
RSC: Research and Surveillance Centre
SAPREL: Secure and Private Record Linkage
